# Insanity

**Published:** 1882-07

**Authors:** 


### INSANITY.

A GENTLEMAN passing along the streets of London not long ago, was suddenly accosted by an entire stranger—"Did you ever thank God that you had never lost your mind?" "Really," replied the gentleman, as soon as he recovered from the surprise which the circumstance excited, "I cannot say that I ever did." "You ought to; for I have lost mine," said the strange interrogator, as he passed rapidly on, and was soon lost in the living tide which ceaselessly flows along the "Strand."

To be a drivelling idiot! to be hopelessly insane! to be feeling after something for a life-time, and never find it; to be for long years in that troubled dream, which in health, before now, although it was but for a moment or two, has caused us to awake, drenched in an agony of perspiration, or found us trembling like an aspen! and yet, reader, that may be your ending! under such circumstances, the lamp of life may go out to you; you may go down to the grave, the universe a blank! We propose telling you how you may avoid it. We will give you no impossible rule, no impracticable recipe, difficult of remembrance, for less than half a dozen words will tell it all—

DON'T DWELL ON ONE IDEA!

Without the *rationale* of this, you perhaps would not remember it twenty-four hours; therefore, in order to impress it on the memory, and save you from so terrible a fate, as a mind in ruins, we will give here the *pathology*, as a doctor would say. *Nutritive degradation*; or if you want the whole idea in a single word, it is "*atrophy*."

Some time ago, we "went to meeting," which, modernized is, "attended church," to hear one of the most scholastic divines of great Gotham. Among other magnificent truths, the speaker declared: "*Anthropomorphism is theopneustic*"! There he left us. As we knew Greek, it was not difficult of remembrance. It took us, however, a good while to dig out the diamond. But we took it in good part, as just then we remembered one of our own definitions of "consumption," in those earlier years when we essayed to be tremendously learned. "*Consumption is the oxydation of the exudation corpuscle.*" That is a fact, to be sure, but it would take a "Philadelphia lawyer" to elaborate it; and we cannot say that a wondering world is any the wiser for either of the grand announcements.

For fear, then, that *nutritive degradation* might meet the fate of all the Capulets, we will abate the top-loftiness of the diction, and come down to the commons.

The brains of all persons dying insane, are withered, as it were, in some portion or other, in the sense that a limb or muscle withers when unused,—withered in a far greater degree than are the brains of those who do not die of insanity. According to the present state of medical knowledge, the whole mass of the brain of a person dying insane, weighs less than it would have done, had the person perished instantaneously, in health.

*Inactivity is destruction throughout the universe of things.* The human body as a whole, or as to any one part, is no exception to that boundless law. The unused arm dwindles to skin and bone. The unused lungs soon weaken, then rot away. The brain comes within the universal law of our physical being, and if unused, perishes before its prime, either in whole or part.

But now we come to the great *phrenological* fact, which only prejudice denies, that the brain is not a unit, but is made up of compartments, each of which is the fountain from which springs the sense, or feeling or sentiment peculiar to it. All men practically believe this essentially, whatever may be their expressed opinions.

The compartments of the brain in the skull, may be appropriately compared to an extensive and well conducted manufactory, with its numberless rooms, in each of which, some one portion of a great machine is made. In one part of our brain, we may say, our mirth is manufactured; in another, our vanity; in another, our pride; and so on; and that brain is in its healthiest state, is the "*best balanced*," in which every room has its proper work, well, fully, and industriously done.

But if one part is worked too much, mischief is the result; or if one part works too little, disorder is inevitable.

If too much mirth is made, the expression leaps from our lips, "He is as funny as a fool;" and we bestow a less complimentary epithet on one who fails to exercise his observant faculties, likening him to the animal which was exactly like a mule, —only more so.

*It is the full, steady, equable exercise of every mental faculty, which is the only infallible guarantee against fatuity.*

Let every man and woman mature this idea well, and steadily guard against one thought, one pursuit, one exclusive employment, one hate, one love, one grief. Blessed is that Providence which seldom sends a single trouble! It is fatherly beneficence which often orders another, to tear the heart away from dwelling on the one great calamity. It is single troubles which craze men. It is not the general student whose mind becomes unbalanced. It is not the man who has a great many irons in the fire at a time; it is not the worker who has more business than he can attend to; it is the man who has leisure to do nothing, it is the man who nurses the one thought wholly, who makes shipwreck of the immortal part. It is the one idea man who is without ballast, and we patronizingly excuse him by saying, "*on every other subject* he is a sensible person."

Asylum statistics force upon us the unexpected truth, that of all classes of inmates, farmers make the largest, in spite of the fabulous health-giving influences of a farming life. Such a result can in no way be accounted for, except in the sameness of thought and pursuit. Another fact, quite unanticipated, is, that in an equal number of New England men, and slaves on southern plantations, the proportion of lunatics is five times greater among the whites; there are five lunatics to one among the negroes; it is because steady concentration in a limited sphere is essential to securing plenty from the stony soil of New England, so barren indeed that multitudes are driven from agricultural pursuits, and in patents and inventions eat out their minds.

Our farmer readers will very naturally inquire, what we would advise as the most perfect safeguard against so lamentable a close of life. Unhesitatingly we respond—Scientific Agriculture; for there is not a quality of the mind which in its far-reachings it will not wake up and energize: for to be properly and most profitably pursued, it makes almost every other science subservient to it. Thus followed, it is the most ennobling of all human pursuits, because it perfects the body, and refines and elevates the mind.

What we have said, therefore, at the commencement of this article, we desire to repeat at its conclusion, with most impressive emphasis—

DON'T DWELL ON ONE IDEA.

